# Indirect costs associated with deaths of children aged 0–14 years from measles in a weak health system and conflict and fragile zone: the case of Somalia

**DOI:** 10.1017/S0950268819001420

**Published:** 2019-08-09

**Authors:** Omar B. Da'ar

**Affiliations:** 1 College of Public Health and Health Informatics, King Saud bin Abdulaziz University for Health Sciences, Riyadh, Saudi Arabia; 2 Graduate School of Professional Studies, St. Mary's University of Minnesota, Minneapolis, MN, USA

**Keywords:** Conflict and fragility, indirect cost of measles, Somalia, the burden of measles

## Abstract

This study recognises periodic outbreaks of measles continue to affect conflict and fragile zones in the least developed countries. This study set out to provide evidence for the indirect costs or economic loss associated with measles-related deaths among children aged 0–14 years in Somalia. Using epidemiologic and economic data, the indirect cost was calculated based on the framework of the World Health Organisation guide of identifying the economic consequences of disease and injury. The baseline indirect cost was computed as the product of discounted future productive years of life lost (PYLL), non-health gross domestic product per capita (NHGDPPC) and the estimated total measles deaths (ETMD). The model was adjusted for conflict and fragility conditions and further extension considered a finite and stable upper limit growth of the instability-adjusted NHGDPPC. To discount future costs, a rate of 3% was applied. Using a ±20% variability assumption of the epidemiologic and economic factor inputs, a sensitivity analysis was conducted to account for uncertainty. In 2015 values, the ETMD of 3723 measles deaths of children aged 0–14 years could decrease non-health GDP of the country by $23.46 million, a potential loss of $6303 per death over the discounted PYLL. The loss would increase by 5.3% when adjusted for conflict and fragility conditions. Assuming growth, the future adjusted loss is expected to be $35.91 million in 2015 values. Girl-child deaths accounted for 51.2% of the burden. Results are robust to the variations in the model inputs, although sensitivity analyses suggest the proportion of total measles deaths and the discount rate accounted for greater uncertainty of the loss than do the proportion of growth and instability assumption. Conflict and fragility accounted for the least uncertainty, perhaps confirming their relative perpetuity in Somalia. Results show significant indirect cost related to measles deaths of children, exacerbated by conflict and fragility. This is an economic burden, but one which the health system, policy-makers, government and other stakeholders should be prepared to colossally discount by collectively taking measles surveillance and security measures now to reduce further deaths in the future.

## Introduction

Global mortality attributed to measles has been on the decline in recent decades [1], significantly contributing to the overall decrease in childhood mortality [2]. Most countries achieved the 2010 global goal of reducing measles mortality by 90% and evidence of measurable progress by 2015 target [3] has recently generated optimism that global eradication is feasible by 2020. Accelerated measles control activities, financial and technical support by high-burden countries have made possible much of the recent progress. In spite of such progress and availability of vaccines, periodic outbreaks of measles continue to appear in many parts of the world including under-vaccinated populations [4] and developed countries [5, 6]. Measles remains a killer of children around the world [7, 8]. Crucial gaps in available data, inadequate surveillance and vital record registrations thwart both the guidance to prevention programmes and eradication of measles [9]. The short-term horizon of 2020 and re-emergence of measles even in traditionally low-burden countries appear to dampen the recent optimism about the disease eradication.

Nowhere is the burden of measles being more felt than in fragile health systems and conflict zones [10]. Somalia has been facing severe development challenges due to political instability and the unending cycle of violence. Conflict and fragility are considered a challenge to sustainable development and 2 billion people live in countries where development outcomes are affected by these conditions [11]. Somalia has remained a fragile state for close to three decades and was ranked the second most fragile state with a score of 114 out of possible 120 [12]. The country remains a fragile state vulnerable to infectious disease outbreaks [13]. In 2012, Somalia was one of the only three countries in the world where immunisation coverage with measles was under 50% and among the few countries still using selected sentinel sites and lacking nationwide surveillance [14].

The motivation for this study is that while combined efforts by the Federal Government of Somalia and the World Health Organisation (WHO) have sought to combat measles in recent years, reported cases increased manyfold. In 2017 alone, there were nearly 20 000 reported cases, four times as many incidences as 2015 and 2016 [15]. Additionally, the motivation for this study comes from the absence of evidence on the extent of economic loss associated with measles-related deaths, especially in conflict and fragile zones. This study set out to provide evidence by estimating the indirect cost associated with measles-related deaths among children aged 0–14 years in Somalia. Specifically, the paper considers the pertinent questions: What is the economic burden or indirect cost associated with measles-related deaths among children aged 0–14 years in Somalia? How do uncertainties about epidemiologic, economic and conflict and fragility conditions affect the magnitude and robustness of the indirect cost associated with measles-related deaths? Estimating the indirect cost of measles in a poor, fragile and conflict-prone country with a weak health care system is important in informing evidence-based interventions, policy and practice that may help reduce mortality.

The rest of the paper is organised as follows: next, the paper presents the methodology in terms of conceptual framework, model specification, computation, discounting and sensitivity analysis. Then, the paper reports baselines and extended results along with sensitivity and robustness checks. Finally, the paper presents the discussion of the results and conclusion.

## Methods

### Conceptual framework

The method involves the estimation of indirect cost associated with measles-related deaths among children aged 0–14 years in Somalia. The paper used a macroeconomic model of cost-of-illness (COI) within the framework of recommendations of reporting economic evaluations according to the WHO guideline for identifying the economic consequences of disease and injury [16]. This paper computed undiscounted productive years of life lost (UDPYLL), discounted productive years of life lost (DPYLL) and conflict and growth-adjusted non-health gross domestic product per capita (^a^NHGDPPC) associated with measles-related deaths. This information was then used to estimate the present value of the non-health gross domestic product loss (NHGDPLoss) associated with measles-related deaths among children aged 01–14 years. Previous studies have used similar approaches to estimate the impact of deaths associated with a disease on non-health components of the future gross domestic product (GDP) [14]. The impact of deaths associated with disease on the non-health components of GDP is the ‘quantity of interest’ when estimating the indirect cost [16–18]. The use of health services or goods does not generate utility or welfare per se [19]. GDP, which is the total value of all marketed final goods and services produced in an economy during a year, is regarded as one of the measures of the market production forgone due to disease death. While medical care and health expenditures actually form part of GDP [18, 20], a more appropriate quantity of interest would be the impact of disease or injury on the non-health components of GDP [17].

### Total non-health macroeconomic loss of measles model and specification

This paper follows the general framework of previous studies [16, 18–20] in measuring the indirect cost by computing the present value of NHGDPLoss due to disease death. In particular, this paper follows the approach of allowing a finite and stable upper limit growth component to NHGDPPC by assuming that NHGDPPC cannot exceed GDP per capita (GDPPC) in any given year in the future [21]. However, while providing a specific context of disease burden in a conflict and fragile health system, the present study augments previous approaches by adjusting the NHGDPPC for conflict and fragility conditions. Conflict and fragility conditions affect the macroeconomic fundamentals of health systems [22].

The NHGDPLoss associated with measles-related deaths among children aged 0–14 years is the product of the total number of DPYLL above the minimum employment age lost, the NHGDPPC and the estimated total number of deaths associated with measles in that age group (ETMD_0–14_). ETMD_0–14_ is calculated from the proportion of total measles deaths of children aged 0–14 years (PTMD_0–14_) out of the total measles deaths reported of the population aged <65 years.

The number of productive years of life lost (PYLL) for children aged 0–14 years was calculated by subtracting the sum of the average age at death (AAD_0–14_) and years remaining to attain the minimum age of employment i.e. the average years lost at the death of a child before attaining the age of employment (YEL) from life expectancy (LE) at birth. In 2015, LE at birth in Somalia was 56.6 years for females and 53.5 for males according to health data we gleaned from WHO updates, World Bank and United Nations for Population [23]; or for simplicity, 57 and 54 years were used. AAD_0–14_ is the simple average age at death for age group 0–14, which equals to 0 plus 14 years divided by 2 (0 + 14/2) = 7. A simple average was used since available evidence of measles deaths did not indicate the distribution by age. The legal minimum age of employment is 15 years according to Article II of the International Labour Organisation (ILO) convention [24]. From AAD_0–14_, YEL is 15 − 7 = 8. Thus, the number of future PYLL equals LE minus 15 years. In 2015, the PYLL for female children aged 0–14 years in Somalia was 42 years, i.e. LE of 57 years minus the sum of AAD (7 years) and YEL (8 years). Similarly, the PYLL for male children aged 0–14 years was 39 years:
1a
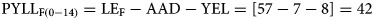

1b
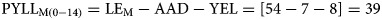
where PYLL, LE, AAD and YEL are defined in the preceding section. The present value of NHGDPLoss to the country due to measles-related deaths among children aged 0–14 years is given by2
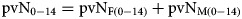
where pvN_0−14_is the present value of total non-health GDP loss due to measles deaths among children aged 0–14 years, disaggregated by gender according to equations (3) and (4):3
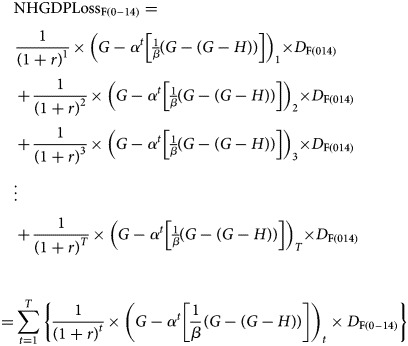
and4
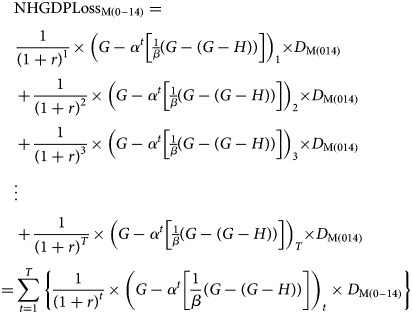
where

- NHGDPLoss_0–14_ is the net present value of conflict- and growth-adjusted total non-health GDP loss due to measles-related deaths among children aged 0–14 years;
- *G* is the GDP per capita;
- *H* is the health expenditure per capita;
- *D* is the estimated total measles deaths (ETMD) that occur among children aged 0–14 years;
- *G-H* is the non-health GDP per capita;
- *α* is a growth adjustment parameter depicting the proportion of non-health GDP per capita to GDP per capita;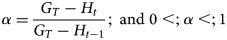, by assumption;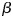 Z is a conflict and fragility-adjustment parameter;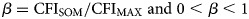, by assumption;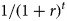 Zis the discount factor;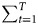 Zdepicts the summation from year *t* to *T*; *t* represents the first year of life lost to measles and *T* is the terminal year, i.e. the final year of all future PYLL per measles death as defined earlier.

### Discounting

The present value of the NHGDPLoss due to measles-related deaths is the sum of NHGDPLoss_F(0–14)_ and NHGDPLoss_M(0–14)_. The NHGDPLoss for each group was estimated by multiplying the total number of DPYLL (which is equivalent to the sum of the discount factors) by the ^a^NHGDPPC and the ETMD_(0–14)_ in that age group. Future costs diminish the more distant in the future they occur, hence society generally value them less than the present costs. This underscores the premise of discounting, which necessitates the need to adjust for the time value of losses occurring in different periods. Discounting future health outcomes have been addressed in previous studies [25, 26]. Discounting future costs and benefits is performed because of time preference, which refers to the desire to realise benefits in the present while deferring any negative effects of doing so [26]. Failure to discount effects even when costs are counted for can lead to inconsistent or misleading results [27]. Failing to discount future costs also has the effect of dampening the present benefits of reducing burden, potentially slowing interventions to be more cost-effective than they would otherwise seem.

### Sensitivity analysis

This paper then conducted a sensitivity analysis to assess the impact of a range of values of a set of epidemiologic and economic factors on the indirect cost associated with measles-related deaths. These factors include the proportion of ^a^NHGDPPC to GDPPC, discount rate, PTDM and conflict and fragility conditions. The existence of a range of values of these factors necessitated the accounting for uncertainty [28, 29], a methodology emphasised in health economic models [30, 31].

## Results

### Baseline results

The 42 and 39 (female and male respectively) UDPYLL among children aged between 0 and 14 years, yielded 23.7 and 22.8 DPYLL after applying a 3% discount rate. This section presents the baseline results without the assumption of instability. The unadjusted non-health GDP per capita (^u^NHGDPPC) in 2015 was $271, which is the GDPPC of $426 less the per capita total health expenditure (PCTHE) of $155. In 2015, the ETMD of the population aged less than 65 years was 8617. The PTMD_0–14_ was 43.2%, translating to ETMD_0–14_ of 3723 (0.432 × 8617). Of these deaths, 50.2% were female translating to 1868 females and 1854 males. At a 3% discount rate, the estimated DPYLL was 23.7 for female and 22.8 for male, respectively. Thus, the baseline present value of NHGDPLoss_F(0–14)_ due to measles-related deaths among female children aged 0–14 years is approximately $12 002 898, which is obtained by multiplying 23.7, the DPYLL, by $271, the ^u^NHGDPPC and 1868, the ETMD_F(0–14)_. On the other hand, the net present value of NHGDPLoss_M(0–14)_ due to measles-related deaths among male children of the same age would be $11 458 553, which is approximately obtained by multiplying 22.8, the DPYLL, by $271, the ^u^NHGDPPC and 1854, the ETMD_M(0–14)_. Together, the baseline results in 2015 values reveal measles deaths among children aged 0–14 years could decrease NHGDP of the country by $23.46 million, representing a potential loss of $6303 per death. Table 1 illustrates the parameterisation of the model used to compute the indirect cost of measles, while Appendix Table A1 reveals the variables and sources of data.

**Table 1.** Computation of measles-related total non-health GDP loss in Somalia

(i)	Estimated total measles deaths <65, ETMD_*T*(<65)_	8617
(ii)	Proportion of total measles deaths, PTMD_*T*(0–14)_	0.432
(iii)	Estimated total measles deaths among children aged 0–14, ETMD_*T*(0–14)_	3723
(iv)	ETMD among females, ETMD_F(0–14)_	1868
(v)	ETMD among Male, ETMD_M(0–14)_	1854
(vi)	Proportion of female population	0.502
(vii)	Proportion of male population	0.498
(viii)	Average age at death for female, AAD_F(0–14)_	7
(ix)	Average age at death for male, AAD_M(0–14)_	7
(x)	Average LE birth, female	56
(xi)	Average LE at birth, male	53
(xii)	Future productive years of life lost for female, PYYLL_F(0–14)_	8
(xiii)	Future productive years of life lost for male, PYYLL_M(0–14)_	8
(xiv)	Gross domestic product per capita, GDPPC	$426
(xv)	Per capita total health expenditure, PCTHE	$155
(xvi)	Unadjusted non-health GDP per capita, ^u^NHGDPPC = (GDPPC − PCTHE)	$271
(xvii)	Conflict and fragility index proportion, CFIP = (114/120)	0.95
(xviii)	Growth and CFI-adjusted non-health GDP per capita, ^a^NHGDPPC = (1/CFIP) × ^u^NHGDPPC	$285
(xix)	A constant growth parameter, ^a^NHGDPPC/GDPPC	0.636
(xx)	Discount rate, *r*	3%
(xxi)	Undiscounted PYLL for female, UDPYYLL__F(0–14)_	42
(xxii)	Undiscounted PYLL for male, UDPYYLL_ _M(0–14)_	39
(xxiii)	Discounted PYLL for female (0–14), DPYLL__F(0–14)_	23.7
(xxiv)	Discounted PYLL for male (0–14), DPYLL__M(0–14)_	22.8
(xxv)	^u^NHGDPLoss_F(0–14)_ = DPYLL__F(0–14_ × ^u^NHGDPPC × D_F(0–14)_ = 23.7 × 271 × 1869	$12 002 898
(xxvi)	^u^NHGDPLoss_M(0–14)_ = DPYLL__M(0–14)_ × ^u^NHGDPPC × D_M(0–14)_ = 22.8 × 271 × 1854	$11 458 553
(xxvii)	^u^NHGDPLoss_F(0–14)_ + ^u^NHGDPLoss_M(0–14)_ = $12 002 898 + $11 458 553 =	$23 461 451
(xxviii)	^a^NHGDPLoss_F(0–14)_ = DPYLL__F(0–14)_ × ^a^NHGDPPC × D_F(0–14)_ = 23.7 × 285 × 1868	$12 634 629
(xxix)	^a^NHGDPLoss_M(0–14)_ = DPYLL__F(0–14)_ × ^a^NHGDPPC × D_M(0–14)_ = 22.8 × 285 × 1854	$12 061 635
(xxx)	^a^NHGDPLoss_F(0–14)_ + ^a^NHGDPLoss_M(0–14)_ = $12 634 629 + $12 634 629 =	$24 696 264
(xxxi)	growth and CFI-adjusted NHGDP loss for female, g_^a^NHGDPLoss_F(0–14)_	$18 378 556
(xxxii)	growth and CFI-adjusted NHGDP loss for female, g_^a^NHGDPLoss_M(0–14)_	$17 526 771
(xxxiii)	g_^a^NHGDPLoss_F(0–14)_ + g_^a^NHGDPLoss_M(0–14)_	$35 905 327

### Results with growth-and instability assumptions

**Fig. 1.**
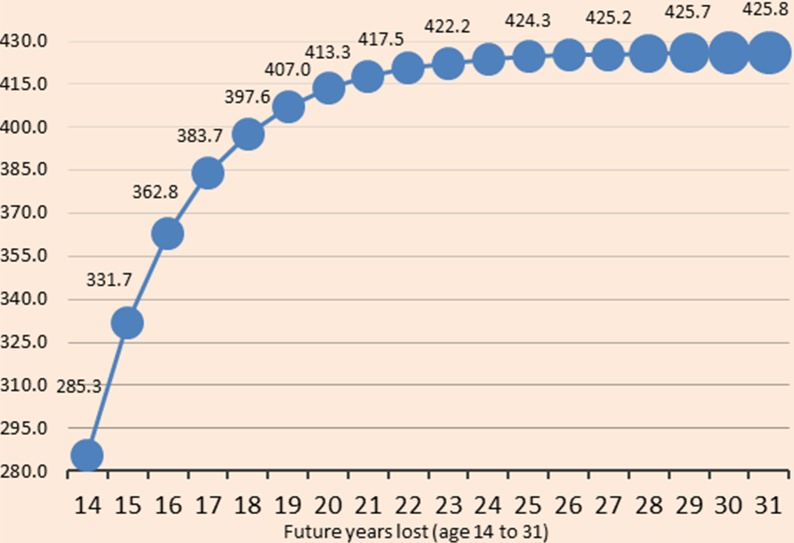
Growth- and CFI-adjusted non-health GDP loss ($ millions) associated with future years of life lost due to measles (from 14 to 31 years).

Figure 1 shows growth- and instability-adjusted non-health GDP loss associated with measles-related deaths increases with PYLL at a decreasing rate. Each additional PYLL results in a smaller increase in the loss since the earlier years of a child's life are more important to future productivity. Conflict and fragility conditions account for part of the bigger marginal losses in the initial future PYLL.

**Fig. 2.**
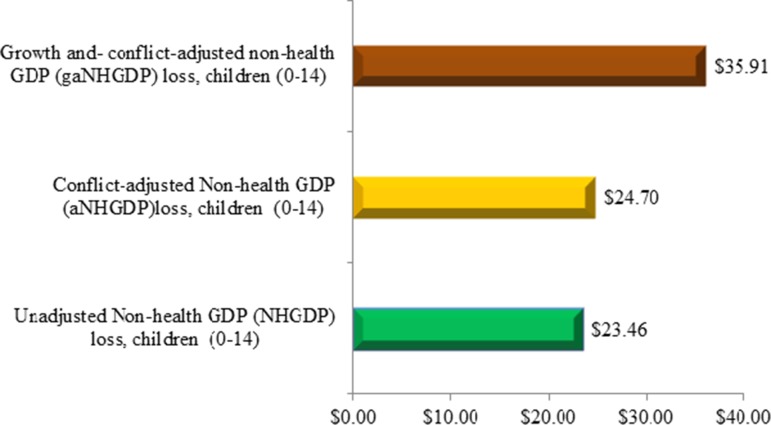
Present value non-health GDP loss ($ millions).

Taking into account conflict and fragility conditions would increase the estimated total loss by 5.3% to $24.70 million. The loss is expected to be $35.91 million over the DPYLL, adjusting the ^a^NHGDPPC for an upper limit modified growth. This translates to a loss of $9645 per death in 2015 value. The upper limit modified growth assumption implies that the NHGDPPC will not increase forever and cannot intuitively exceed GDPPC in any given year in the future. Figure 2 depicts the baseline results with conflict and growth adjusted values.

**Fig. 3.**
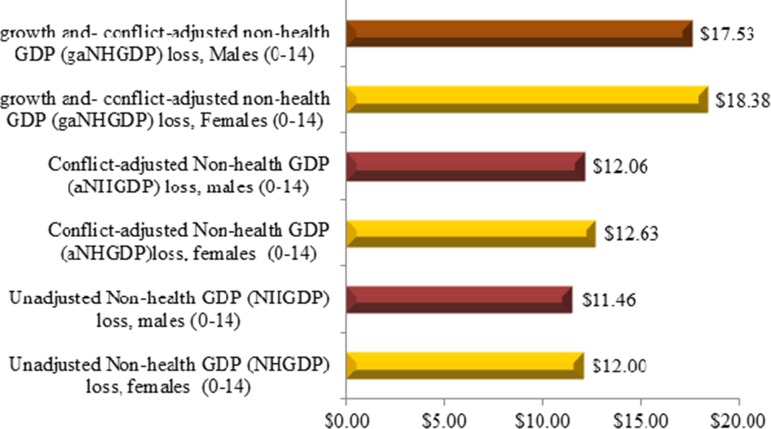
Present value non-health GDP loss ($ millions) by gender.

Disaggregating the NHGDPLoss by gender reveals that female children aged 0–14 years would approximately account for 51.2% of the indirect cost related to measles deaths (Fig. 3).

### Sensitivity results

Table 2 depicts the model factor input assumptions with baseline values and variability of ±20%.

**Table 2.** Baseline parameter assumption values

Factor	20% Reduction	Base scenario	20% Increase
Discount rate	2.40%	3.00%	3.60%
Growth-adjustment factor of NHGDP per capita	0.54	0.67	0.8
Proportion of total measles deaths (PTMD_0–14_)	0.35	0.43	0.52
Conflict and fragility index	91.2	114	120

Table 3 shows a 20% reduction in the discount rate, the proportion of ^a^NHGDPPC to GDPPC and conflict and fragility conditions increased the indirect cost by 10.8%, 1.1% and 1.3% to $39.79, $36.31 and $36.4 million, respectively. These increases were up from the baseline case of $35.91 million. However, a 20% reduction in the PTMD_(0–14)_ decreased the indirect cost to $28.73 million, representing a fall of 20% down from the baseline case of $35.91 million. Table 3 also shows that a 20% increase in the discount rate, the proportion of ^a^NHGDPPC to GDPPC and conflict and fragility conditions decreased the indirect cost to $32.56, $35.02 and $35.81 million, respectively, down from the baseline case of $35.91 million. However, a 20% increase in the PTMD_(0–14)_ symmetrically increased the indirect cost by 20% to $43.09 million, up from the baseline case.

**Table 3.** Sensitivity and adjusted sensitivity of indirect cost associated with measles-related deaths ($000’)

		20% Reduction		20% Increase		Range
Factor	Baseline	20% Reduction	Change 1	20% Increase	Change 2	Abs(Change 1 − Change 2)
Discount rate	$35 906.99	$39 786.33	$3879.34	$32 563.47	−$3343.52	$7222.86
Growth-adjustment factor of NHGDP per capita	$35 906.99	$36 312.58	$405.59	$35 021.93	−$885.06	$1290.65
Proportion of total measles deaths (PTMD_0–14_)	$35 906.99	$28 725.59	−$7181.40	$43 088.39	$7181.40	$14 362.80
Conflict and fragility index	$35 906.99	$36 400.23	$493.24	$35 808.34	−$98.65	$591.89

Finally, Table 3 shows adjusted sensitivity, which essentially is the relative and absolute changes (ranges), associated with a ±20% variability of the parameter assumptions. A 20% reduction in discount rate, the proportion of ^a^NHGDPPC to GDPPC and conflict and fragility conditions would add to the baseline total loss approximately $3.88 million, $405 590 and $493 240 respectively, while a 20% increase in these factors reduces the total loss by $3.34 million, $885 060 and $98 650 respectively, during the same period. Allowing the PTMD_(0–14)_ to vary in a similar fashion would produce the opposite effect. For example, a 20% reduction in the PTMD_(0–14)_ would decrease the total loss by approximately $7.18 million while a 20% increase in this factor would symmetrically add the total loss same amount.

The foregoing sensitivity analyses show that the NHGDPloss associated with measles-related deaths would change when the variability of the different model factor input assumptions are taken into consideration. However, the changes do not much influence the actual estimates in relation to the baseline value, suggesting that the results are robust to the different model factor input changes.

**Fig. 4.**
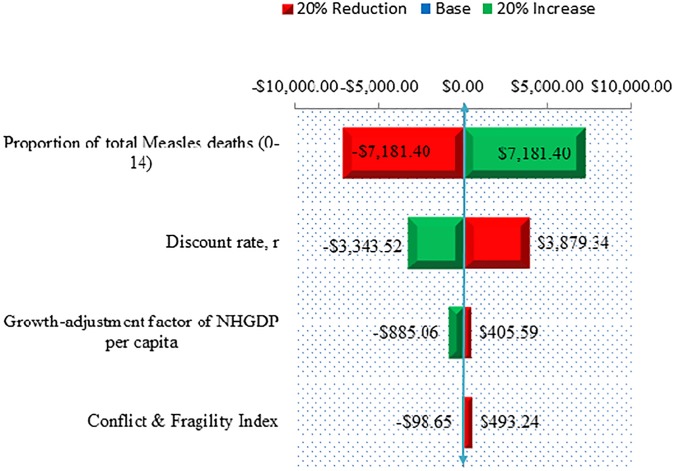
A Tornado Diagram depicting the sensitivity of NPV Non-Health GDP per Capita loss ($000’) with respect to epidemiologic and economic factors.

Figure 4 shows a Tornado diagram depicting the sensitivity of the NHGDPLoss with respect to epidemiologic and economic factors. The PTMD_(0–14)_ and the discount rate would account for much of the variability of the loss to the economy. However, less uncertainty would be attributable to instability and growth adjusted assumptions in the health system and macroeconomic fundamentals and conflict and fragility conditions.

## Discussion

Setting out to provide evidence in Somalia, this study estimated the indirect cost associated with measles-related deaths among children aged 0–14 years. Generally, the literature shows that measles continues to affect African countries [32, 33]. The disease remains a killer among vaccine-preventable diseases, especially among children of developing countries [34, 35]. Additionally, a recent systematic review of the epidemiologic and economic burden of measles, mumps, pertussis and varicella in Germany suggested that there was still considerable morbidity due to childhood diseases [36]. The present study estimation of a significant indirect cost associated with measles-related deaths may be a clear pointer that the disease continues to affect Somalia, especially among children 14 years and younger, robbing off their future productivity and contributions to society. The findings support outcomes of previous studies which reported that measles complications and deaths were disproportionately borne by underage children [37, 38]. The results also relate to the continued global burden of measles in terms of incidence even in developed countries in the European and Western Pacific Regions [39, 40] and in the United States [40, 41]. The economic burden of measles in these countries, however, mostly related to direct healthcare costs [43–47].

The results further suggest that the estimated economic loss due to measles-related deaths would increase with conflict and fragility conditions over the discounted future PYLL, adding to Somalia's both short-term and long-run economic burden. While the present study provides a different context and considers the additional assumption of conflict and fragility conditions, the results are not only consistent with the indirect and direct cost of measles [48, 49], but also congruous to findings of studies of other high burden infectious diseases [20].

Previous studies examined gender differentials with regards to measles burden. A world review of gender differences in measles mortality showed that females had higher mortalities than males even though they may have generally lower mortalities than males in all ages [50]. The results suggest that measles-related deaths of girls aged 0–14 years would account for slightly more than half of the loss (51.2%).

To account for uncertainty in data and robustness of results, health economics literature emphasises the need for sensitivity analysis [28–31]. The present study suggests the model and results are robust although somewhat sensitive to changes in the epidemiologic and economic factors, affirming the need for sensitivity analysis. The PTMD_0–14_ and the discount rate were found to account for much of the variability of the economic loss. Conflict and fragility conditions were associated with less variability, perhaps due to the increasing regularity of such situations in Somalia, a country with weak health and economic systems. The analyses of this paper make a unique contribution to evidence of the economic burden, especially the indirect cost associated with measles-related deaths of children in Somalia. The analyses make a case for estimating and quantifying the total NHGDP loss associated with DPYLL due to measles while adjusting for conflict and fragility conditions in a country with weak health and economic systems. Measuring the indirect cost of measles in this manner is important as it may inform evidence-based interventions that may potentially reduce mortality.

It is important to note several limitations, however. It should be noted that the analysis of this paper was limited to the indirect cost of measles. The analysis did not include direct healthcare costs of measles, making it a partial evaluation. This means that the estimated non-health loss was only related to the value of resource lost due to measles deaths. Potential morbidity costs were not included because of data limitation, implying the absence of some indirect cost relating to the value of future losses in productivity or potential illness or disabilities that may limit the full contributions of people to society. While the estimated measles-related economic burden may inform the need to increase allocation towards prevention or direct healthcare management, it is limited in determining how resources are to be distributed [51]. Thus, this study is limited in informing public health priority settings, in part because of the absence of costs of alternative interventions that could prevent measles-related morbidity and mortality [52, 53]. Finally, there is underreporting of measles in Somalia since the country has fewer than 50% immunisation coverage and uses selected sentinel sites, lacking nationwide surveillance. Consequently, the economic burden captured and reported in this study may be conservative and should be interpreted as such.

## Conclusion

In conclusion, the present study's adjustment for conflict and fragility conditions extends the COI approaches and framework of reporting economic evaluation, especially the estimation of economic consequences of disease and injury. The results suggest a significant indirect cost associated with measles-related deaths of children aged 0–14 years in Somalia. The findings underscore the extent of the additional burden of conflict and fragility conditions on measles-related indirect cost in a weak healthcare system, which may point to underlying challenges of reducing mortality.

Given the suggestion that the PTMD_0–14_ was attributed to the greatest variability in the estimated loss and the importance of reducing measles mortalities to health policy expert and practitioners, continued concerted effort involving systemised healthcare and disease control programmes are paramount. Such programmes should include deliberate nationwide surveillance to increase universal coverage of vaccinations by the healthcare system and/or government and non-government actors. Since conditions of conflict and fragility add up to the economic burden of measles, there needs to be simultaneous concerted to promote security and anti-poverty programmes.

The present value of the estimated indirect cost was robust although it exhibited sensitivity to the variability of the discount rate. While the cost is a future economic burden, it is one which the health system, policymakers, government and other stakeholders should be prepared to colossally discount. Thus, any intervention to control measles could not be timelier as collective actions such as disease surveillance and security measure could be taken now to reduce measles deaths of children 0–14 years in the future. Otherwise, future measles-related costs might be colossal. The society must today place significant value on future benefits to be realised some years to come.

Further research is required to study the full cost of measles, including direct cost. In addition, there is an urgent need for investigating the capacity of the national and local health systems in discounting future burden of measles by responding to periodic outbreaks.

## Data

Data were collected from various sources, but inputs used can be availed upon request.

## Appendix

**Table A1.** Data inputs and sources

	Variable	Data sources
1	Somalia average life expectancy at birth, female and male	World Health Organization (WHO), World Bank, United Nations for Population, World life expectancy country profiles
2	Undiscounted PYLL female and male (0–14 year olds)	Author computation from data sources
3	Discounted PYLL female and male (0–14 year olds)	Author computation from data sources
4	Total measles deaths female and male	Health profile Somalia – World life expectancy country profile
5	Proportion of total measles deaths (0–14 year olds)	Author computation data sources and age structure
6	Proportion of female and male population	World Bank
7	Average age at death for 0–14 year olds	Author computation from data sources
8	Gross domestic product per capita (GDPPC)	World Bank
9	Health expenditure per capita (HEPC)	World Bank
10	Non-health expenditure GDP per capita (NHGDPPC)	Author computation from data sources
11	Growth adjustment parameter	Author computation
12	Conflict and Fragility Index	Fund for Peace
13	Discount rate (*r*)	World Health Organization (WHO)
